# Report From Bolsonaro’s Brazil: The Consequences of Ignoring Science

**DOI:** 10.1177/0020731420968446

**Published:** 2020-10-25

**Authors:** Alvaro J. Idrovo, Edgar F. Manrique-Hernández, Julián A. Fernández Niño

**Affiliations:** 1 Public Health Department, Universidad Industrial de Santander, Bucaramanga, Colombia; 2 Public Health Department, Universidad del Norte, Barranquilla, Colombia

**Keywords:** pandemics, politics, public information, science

## Abstract

Currently, the fast spread of COVID-19 is the cause of a sanitary emergency in Brazil. This situation is largely due to President Bolsonaro’s denial and the uncoordinated actions between the federal and local governments. In addition, the Brazilian government has reported that it would change its method of sharing information about the pandemic. On June 6, 2020, the presentation of accumulated cases and deaths was stopped, and the Supreme Court of Brazil determined that the federal government should continue to consolidate and disseminate the accumulated figures of cases and deaths. However, doubt about the transparency of the data remained. We used data reported by the government from Situation Reports 38–209 of the World Health Organization to assess the Benford’s law fulfillment as an indicator of data quality. This rapid evaluation of data quality during the ongoing COVID-19 pandemic in Brazil suggests that the Brazilian public health surveillance system had an acceptable performance at the beginning of the epidemic. Since the end of June, the quality of cumulative death data began to decrease and remains in that condition as of August 2020. A similar situation has existed since August, with the data of accumulated new cases.

On February 25, 2020, the first case of SARS-CoV-2 infection was diagnosed in Brazil,^1^ the first country with cases in Latin America. Currently, the fast spread of this infection is the cause of a sanitary emergency in various Brazilian regions. Up to August 16, 2020, 3,275,520 cases and 106,523 deaths by COVID-19 were reported by the World Health Organization (WHO) in the final daily COVID-19 Situation Report. These figures are close to the reports included in the interactive, web-based board by the Center for Systems Science and Engineering, Johns Hopkins University,^2^ where 3,501,975 cases and 112,304 deaths were reported on August 20, 2020. Thus, Brazil is the second country, after the United States, with the most cases and deaths in the world. This tragedy is largely due to President Bolsonaro’s denial and the uncoordinated actions between the federal and local governments.^3,4^ As in other countries, underreporting of mortality was a frequent complaint and could be quickly estimated using various methodologies.^5,6^ However, among Brazilians, doubt arising from distrust in the data presented by the government continued to grow (https://coronavirus.jhu.edu/).

As another manifestation of presidential denial, the Brazilian government at the beginning of June reported that it would change the method of sharing information about the pandemic. On June 6, 2020, the presentation of accumulated cases and deaths was stopped, and the following day, only new cases were reported, without providing information on accumulated cases on the official website (https://covid.saude.gov.br/). According to the federal government, this would facilitate the communication of data to the public. However, this was understood as a way of hiding information about the real magnitude of the adverse effects,^5^ which responded to the political interests of the government headed by President Bolsonaro.^7^ As a response to this lack of transparency, the media organizations *O Estado de S. Paulo*, *Folha de S. Paulo*, *O Globo*, *Extra*, *G1*, and *UOL* decided to organize a network that would allow them to continue to have timely, quality data.

Because of complaints about this change in reports, both inside and outside the country, the Supreme Court of Brazil determined on June 8, 2020, that the federal government should continue to consolidate and disseminate the accumulated figures of cases and deaths. This was accepted by President Bolsonaro, and accumulated cases and deaths began to be reported again. However, the crisis within the Ministério da Saúde is evident, and it is reflected in the ministerial changes that occurred this year. Luiz Henrique Mandetta, a children’s orthopedic doctor, was fired by the president on April 16, 2020. Then there was Nelson Luiz Sperle Teich, a medical oncologist, who defended the lockdown as a measure to control the pandemic and resigned on May 15, 2020. Since May 16, 2020, Army General Eduardo Pazuello has held the position of Minister of Health without having experience in the health sector. A key question is whether the data that Brazil had been reporting were of low quality. In this report, we attempted to resolve this question.

## Methods

To obtain evidence on the data quality of the Brazilian public health surveillance system, we used data reported by the government from Situation Reports 38–209 on the WHO website. This represents the period between February 27 and August 16, 2020. The analysis included a graphical representation of the cumulative number of confirmed cases and deaths by COVID-19 and the assessment of Benford’s law fulfillment.

When the data follow the distribution of the first digits (Benford’s law),^8^ that is a strong indication that the surveillance system is performing adequately. The influenza A(H1N1) pandemic was the first time Benford’s law was used to explore the data quality of public health surveillance.^9^ Subsequently, it was used to evaluate the public health surveillance of Paraguay during the dengue epidemic (2009–2011)^10^ and the Zika epidemic in the Americas.^11^ Recently, it was used to explore the Chinese and Colombian COVID-19 data.^12,13^

According to Benford’s law, the most frequent digits in situation reports should be ones (30.103%), followed by the other digits in order from 2 to 9 (17.609%, 12.494%, 9.691%, 7.918%, 6.695%, 5.799%, 5.115%, and 4.576%, respectively).^8^ Brazilian surveillance data of COVID-19 (cumulative cases and deaths) were evaluated according to how closely they followed the distribution of Benford’s law using a log likelihood ratio test. Given the chaos generated by the presidential order, a first analysis covered the period to June 8, 2020, and a second analysis covered the period August 16, 2020. These analyses were conducted with Stata 14 statistical software (Stata Corporation, USA), using the *digdis* macro developed by Ben Jann (ETH Zurich).

## Results

**Figure 1.**
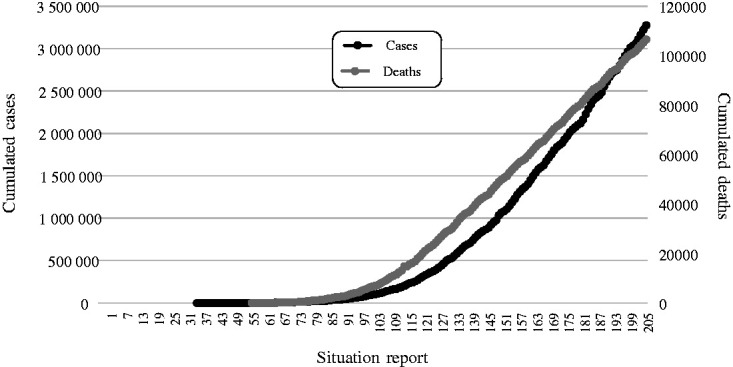
Cumulated cases of COVID-19 in Brazil until August 16, 2020, according to the Brazilian government (as reported to the World Health Organization).

The Brazilian government reported the first case to WHO on February 27, 2020, and the first death on March 19, 2020. Figure 1 shows the number of cumulated confirmed cases and deaths by COVID-19. The similarity between the 2 curves, despite their different magnitudes, is evident. However, in the last dates, a cross between the 2 lines was observed, suggesting that the ratio between new cases and deaths had changed.

Results of the analyses, which evaluate how closely the data of cumulated confirmed cases and deaths followed the distribution of Benford’s law, are in Table 1. As can be seen, during the first 2 months of the pandemic in Brazil, the quality of data fluctuated; sometimes Benford’s law was fulfilled (higher *p*-values) and sometimes it was not (lesser *p*-values). This is common at the beginning of epidemics. It is evident that since the end of June, the quality of cumulative death data began to decrease and remained in that condition until August 2020. Something similar has happened since August, with the data of accumulated new cases.

**Table 1.** Fulfillment of Benford’s Law of Brazilian Public Health Surveillance System of COVID-19 (Cumulative Confirmed Cases and Deaths) Until Selected Dates.

WHO’s Situation Reports		Log Likelihood Ratio ( *p* Value)	
(Since 38)	Last Date	Cases	Deaths
Until 70	March 30	0.0217	0.2717
Until 80	April 9	0.1304	0.4777
Until 90	April 19	0.0249	0.5239
Until 100	April 29	0.0998	0.3975
Until 110	May 9	0.3786	0.6666
Until 120	May 19	0.1926	0.5819
Until 130	May 29	0.0953	0.3221
Until 140	June 10	0.2543	0.5469
Until 150	June 18	0.7188	0.3229
Until 160	June 28	0.8889	0.0563
Until 170	July 8	0.9143	0.0360
Until 180	July 18	0.6628	0.0144
Until 190	July 28	0.2729	0.0158
Until 200	August 7	0.0206	0.0206
Until 209	August 16	0.0259	0.0106

**Figure 2.**
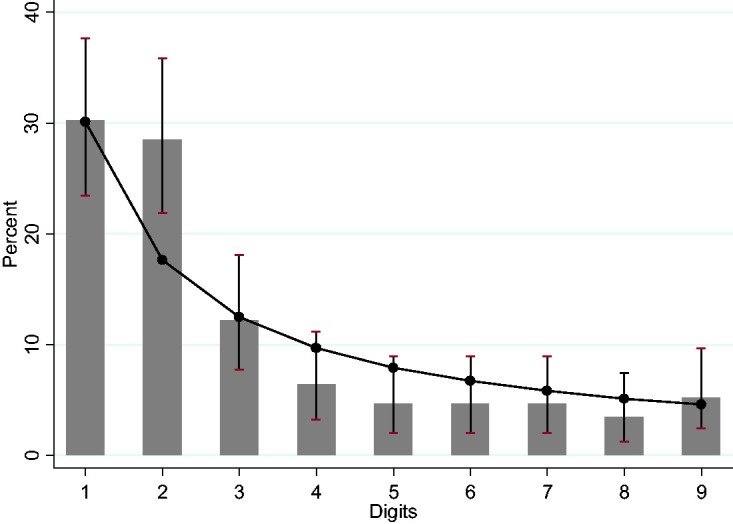
First-digit frequencies for the Benford distribution of daily reports of cumulated cases in Brazil until August 16, 2020. Bars are the empirical data and markers, with their respective lines, the Benford distribution, and confidence intervals.

**Figure 3.**
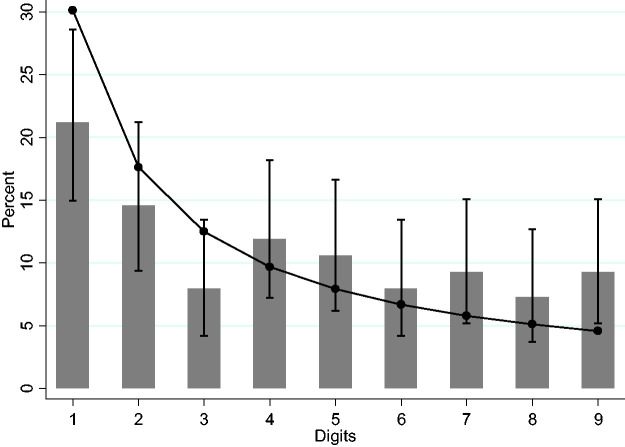
First-digit frequencies for the Benford distribution of daily reports of cumulated deaths in Brazil until August 16, 2020. Bars are the empirical data and markers, with their respective lines, the Benford distribution, and confidence intervals.

Figure 2 shows the distribution of the first digits of cumulated new cases until August 16, 2020. It is notable that the number 2 as the first digit occurs much more frequently than expected (*x*^2^, *p*-value = 0.0004). In relation to accumulated deaths, Figure 3 shows that instances of the number 1 as the first digit are few (*x^2^*, *p*-value = 0.0165), and instances of the number 9 as the first digit are many (*x*^2^, *p*-value = 0.0106).

## Discussion

Before interpreting the findings, it is important to know that quality data during an epidemic do not prevent underreporting, because this is a common phenomenon in health crises and reporting can be verified later. This evaluation of data quality during the ongoing COVID-19 pandemic in Brazil suggests that the Brazilian public health surveillance system had an acceptable performance, with some ups and downs, until June. Since July, the accumulated mortality data—and, since August, the accumulated new cases—do not fulfill Benford’s law, suggesting poor data quality. An explanation of these findings corresponds to a hypothesis that could be explored in other studies. Perhaps the Ministry of Health was in crisis during the management of the pandemic, with the most notable political evidence being the change in ministers.

This is surprising because during the influenza A (H1N1) pandemic, performance was acceptable,^9^ suggesting that Brazil had a good surveillance system. Unfortunately, in both cases, mortality has been high.^9,14^ These results of public health surveillance are consequences of the disorder in the management of the pandemic, where there are difficulties with the actions of field epidemiology and diagnostic laboratories. As always, deaths are more difficult to hide, so Benford’s law showed better results. However, even if these information limitations were greater, they do not justify attempts to decrease their public availability in Brazil.

Open data in public health is not only a technical or legal standard, nor a technology infrastructure; open data is primarily a public policy. It is a decision by a government to generate, consolidate, analyze, and divulge information to ensure that data collected during public health emergencies, such as pandemics, are accessible to the appropriate authorities and society and to consequently contribute to timely decision-making at various levels.^15^ Open and transparent data has been recognized as an important element for an adequate response to the COVID-19 pandemic. It is an important element to prevent panic and confusion among members of society^16^ and serves as the primary knowledge source to support better decisions about daily activities. One of the most studied examples is South Korea, where detailed data on diagnosed cases and deaths was quickly available to the public. This, together with various technological strategies, allowed the construction of a robust public health surveillance system with rapid response,^17^ which is related to the successful management of the pandemic.

According to the United Nations Educational, Scientific and Cultural Organization (UNESCO),^18^ Brazil has the largest number of institutional open-access policies (n = 16) in Latin America. In addition, it is the country in South America with the largest scientific production in health sciences and great development in public health. These contrast with the results of the Intelligent Citizenship Foundation (Fundación Ciudadanía Inteligente), which reported that among Latin American countries, Colombia, Chile, Mexico, and Peru have better data transparency than Brazil.^19^

However, Bolsonaro’s stance, not only in relation to the pandemic, but in several issues of public health and environmental policy, has been characterized as an antiscientific discourse, where scientific information, even official information from his own government, is neglected when it contradicts his political and ideological views or their political interests.^20^ Decisions to hide data could not only affect neighboring countries, but also sabotage the performance of their own ministers and the governments of the states of their countries, putting millions of vulnerable people at risk. Transparency in public health data is not only technically useful for the organization of the response and for international cooperation, but also allows for citizen oversight, accountability, and stronger community participation. Furthermore, the lack of transparency in the data favors corruption, affects democratic mechanisms, prevents the evaluation of policies,^21^ and allows for consolidation of alternative discourses, such as those of the Bolsonaro government.

All public health data has limitations, and when they are collected during a crisis such as a pandemic and reported in real time, they have several technical limitations. However, transparency allows for identifying problems in the available data and for improving them progressively. Under no circumstances should concealment or restriction of data be justified in its limitations, because doing so may cause reputational harm, instill institutional mistrust, and affect the public health emergency response. The role of political power should be to consolidate scientific information and guide decisions considering the economic and sociocultural dimensions, not to torpedo the management of knowledge, which seems to be what the Bolsonaro government is seeking.
